# The effect of 8 plant extracts and combinations on post-prandial blood glucose and insulin responses in healthy adults: a randomized controlled trial

**DOI:** 10.1186/s12986-020-00471-x

**Published:** 2020-07-06

**Authors:** David J. Mela, Xiu-Zhen Cao, Rajendra Dobriyal, Mark I. Fowler, Li Lin, Manoj Joshi, Theo J. P. Mulder, Peter G. Murray, Harry P. F. Peters, Mario A. Vermeer, Zhang Zhang

**Affiliations:** 1 Unilever R&D, Wageningen, the Netherlands; 2 Unilever R&D Shanghai, Shanghai, China; 3 Hindustan Unilever Research Centre, Mumbai, India; 4 Unilever R&D, Sharnbrook, UK; 5 Unilever R&D, Bangalore, IN India

**Keywords:** Glycemic response, Dietary intervention, Breath hydrogen, Enzyme inhibitors, Glucose transporters

## Abstract

**Background:**

Lower post-prandial glucose (PPG) and insulin (PPI) responses to foods are associated with reduced diabetes risk and progression. Several plant extracts have been proposed to reduce PPG or PPI by inhibiting enzymes or transporters involved in carbohydrate digestion and uptake. This study evaluates a range of such extracts, consumed with a carbohydrate load, for their effects on PPG, PPI and indicators of (gastrointestinal) tolerance.

**Methods:**

Interventions were extracts of mulberry fruit (MFE, 1.5 g), mulberry leaf (MLE, 1.0 g), white bean (WBE, 3.0 g), apple (AE, 2.0 g), elderberry (EE, 2.0 g), turmeric (TE, 0.18 g), AE + TE, and EE + TE. Each of these 8 individual extracts or combinations were added to a rice porridge containing ~ 50 g available carbohydrate (control). In a within-subject (randomised, balanced incomplete block) design, individual subjects received the control and a subset of 4 of the 8 extracts or combinations. Participants were 72 apparently healthy adults (mean [SD] age 31.2 [5.5] yr, body mass index 22.1 [2.0] kg/m^2^). The primary outcome was the percentage change in 2-h PPG (positive incremental area under the curve) relative to control. Secondary measures were the 2-h PPI response, 7-h breath hydrogen, measures of gastrointestinal discomfort, and urine glucose.

**Results:**

In the 65 subjects who completed the control and at least one intervention treatment, additions of AE, MFE and MLE produced statistically significant reductions in PPG vs control (*p* < 0.05; mean effect − 24.1 to − 38.1%). All extracts and combinations except TE and WBE significantly reduced PPI (*p* < 0.01; mean effect − 17.3% to − 30.4%). Rises in breath hydrogen > 10 ppm were infrequent, but statistically more frequent than control only for MLE (*p* = 0.02). Scores for gastrointestinal discomfort were extremely low and not different from control for any treatment, and no glucosuria was observed.

**Conclusions:**

Additions of AE, MFE and MLE to rice robustly reduced PPG and PPI. EE significantly reduced only PPI, while TE and WBE showed no significant efficacy for PPG or PPI. Breath hydrogen responses to MLE suggest possible carbohydrate malabsorption at the dose used, but there were no explicit indications of intolerance to any of the extracts.

**Trial registration:**

ClinicalTrials.gov identifier NCT04258501. Registered 6 February 2020 - Retrospectively registered.

## Introduction

Lower post-prandial blood glucose and insulin responses (PPG and PPI, respectively) are associated with a lower risk of development and progression of diabetes and cardiovascular diseases in healthy populations as well as those with (pre-)diabetes [1–4]. A large volume of research has considered specific components or compositions of foods and diets that could offer cost-effective and well tolerated approaches to reduce PPG and PPI [5]. One widely-known approach is to change the composition and characteristics of fibres or starches in carbohydrate-rich foods [6]. However, this has potentially large impacts on product physical and sensory (handling, textural, flavour) attributes, and is only suitable for certain product formats.

An alternative approach is to identify ‘functional’ ingredients, efficacious at low levels for reducing PPG and PPI when consumed in or with foods and beverages containing significant amounts of glycemic carbohydrates. An example would be ingredients that act pre-absorptively, as natural sources of inhibitors of enzymes (α-amylase, α-glucosidase) or transporters (Sodium-glucose linked co-transporter 1 [SGLT1], Glucose transporter 2 [GLUT2]) involved in carbohydrate digestion and uptake. These gut-based mechanisms have previously been proposed as potential intervention targets for managing rates of glucose availability from foods [7–12]. A variety of plant extracts or combinations have been reported to have in vitro activity against these (and other) targets, in some cases also having data indicating clinical efficacy for glycemic control [13]. Many of these may be available as supplements or used in traditional medicine. However, not all of these extracts are available as well-characterized, food-grade materials that are realistically feasible (in terms of safety and regulatory requirements, sensory acceptability, stability, etc) for wide-scale use in commercially manufactured foods and beverages. Furthermore, the efficacy and tolerability of these have rarely been directly evaluated under well-controlled and standardized clinical test conditions with a healthy population and consumer-relevant test product.

While emphasis is generally placed on PPG, it is also seen as desirable to achieve this without increasing PPI. The European Food Safety Authority [14] has stated that reduced PPG may be considered a beneficial physiological effect “… as long as insulin responses are not disproportionally increased”. Doubt over the long-term health benefit of stimulating insulin release per se as a route to reducing PPG is indicated by data from Nateglinide, a drug stimulating insulin release, that was found ineffective in reducing the transition from pre-diabetes to diabetes [15]. Reducing PPG without increasing PPI also provides some additional indication that effects can be attributed to the primary expected mechanism of reduced glucose entry rates, rather than enhanced disposal.

A substantial inhibition of carbohydrate digestion or absorption could, however, also lead to malabsorption and risks of gastrointestinal side-effects and poor consumer tolerance [16]. It is therefore important to assess possible indicators of this, such as breath hydrogen (H_2_) and reported gastrointestinal symptoms. In addition to pre-absorptive effects, there are also possible post-absorptive effects if dietary components are absorbed in a bioactive form. For example, if agents that inhibit glucose uptake via SGLT1 in the gut are absorbed, they could act systemically to inhibit glucose re-uptake in the renal tubules via the SGLT1 or structurally similar sodium-glucose linked co-transporter 2 [SGLT2] present there [17]. Inducing glucosuria by such a mode of action may be viable for the pharmaceutical management of diabetes, but can contribute to an increased risk of urinary tract infection, which would not be an acceptable side-effect for food ingredients in widespread consumption by the general public.

As a first step in assessing plant extracts with potential to be used as food ingredients with substantiated PPG or PPI efficacy, we identified several commercially available extracts or combinations of these with reported clinical and/or in vitro evidence for potential efficacy, assessed their bioactivity in vitro, and tested their efficacy and tolerability in human subjects using well-characterized materials under standardized conditions.

## Method

The trial was designed to test efficacy and tolerance using eight plant extracts or combinations of these for reducing the PPG and PPI responses to a carbohydrate load, relative to a control with no extract added. All subjects received the control product and a subset of 4 of the 8 extracts or combinations, in a within-subject (balanced incomplete block) design. The clinical phase was executed between 24 Nov 2011 (first subject signed informed consent) and 6 Jan 2012 (last subject last visit) at Lambda Therapeutics Research Ltd. (LTRL), India.

### Participants

Potential participants were recruited from a pre-existing database of healthy volunteers at LTRL. Overweight and older subjects were excluded to minimize the risk of recruiting individuals with (undiagnosed) impaired glucose tolerance. Subjects were therefore selected in the age range 20–50 yr, with a body mass index (BMI) in the “normal” range (18-25) according to the World Health Organization guidance [18]. The complete inclusion and exclusion criteria are described in the Supplementary Material, Table S1. Subjects were furthermore screened for and included on the basis of a positive test for lactose-intolerance. This criterion was adopted for the trial because the putative active components of two of the interventions (phloridzin and cyanidin-3-glycoside in apple and elderberry extracts, respectively) are flavonoid glycosides, and converted to their aglycone forms by the action of intestinal beta-glycosidases, primarily lactase-phloridzin hydrolase (LPH) [19, 20]. The glucoside and aglycone forms of flavonoids may express differing specificity for inhibition of sodium-dependent (eg SGLT1) and sodium-independent (eg GLUT2) glucose transport mechanisms [8]. Therefore, higher LPH activity could induce higher rates of flavonoid glucoside hydrolysis [20], and alter the apparent efficacy of AE and EE. In order to minimize this potential source of variance at this stage of the research, we selected for subjects with a uniformly low LPH activity as manifested by lactose intolerance.

Potential participants were screened in 2 sessions prior to the start of the intervention. The full study flow schedule for volunteers is detailed in the Supplementary Material, Table S2. Instructions, self-report interviews, and data collection were all undertaken in the native language of participants.

### Design and allocation to treatments

The study had a balanced incomplete block design, with each subject randomly allocated to a treatment sequence in which they received the control product and a subset of 4 of the 8 test extracts, as one test product per week over 5 weeks. Subjects entered the trial in one of 4 intake cohorts with equal numbers of males and females. The randomization scheme was computer generated at the test site, and not accessible to personnel involved in the collection, monitoring, revision, or evaluation of adverse events, nor to clinical laboratory or other personnel who could have an impact on the outcome of the study, until after the end of the clinical and analytical phases. Persons involved in the preparation of test products were not involved in the rest of the study. Other study team personnel and subjects and were blinded to the identification of specific treatments but the products varied somewhat in colour or flavour.

### Interventions

The test ingredients were selected from commercially available food-grade extracts with proposed enzyme or transporter inhibitor activity, potentially feasible for use as commercial food ingredients. They were identified and selected for inclusion on the basis of available literature showing clinical or pre-clinical indications of efficacy, potential to have a sustainable supply chain, knowledge of putative active components (thus ability to set and test specifications), forecast of safety and regulatory acceptability (for clinical testing and eventual market presence), and suitability for commercial food processing and formats. Doses were based on recommendation or efficacy data from the supplier or published literature where available, or alternatively the maximum dose approved for acute human testing following a safety assessment by the Unilever Safety and Environmental Assurance Centre.

The Supplementary Material text provides brief background descriptions of each selected extract, details of the analytical specifications, and results of in vitro tests of bioactivity of each extract for its putative target (digestive enzymes or glucose transporters). All of the extracts were acquired from commercial suppliers; none were products of the company sponsoring the research. The dose of each extract was not adjusted for body weight, because subjects served as their own controls, and the putative mechanisms of action were all pre-absorptive (in the gut, not systemic, so no clear basis for adjusting doses for a systemic dilution effect of body size).

The extracts and combinations, and doses tested here were:

1. Mulberry fruit extract, 1.5 g (MFE; batch No. MF-DC-KQ, Draco Natural Products Inc., San Jose CA, USA), containing 0.5% (w/w) of the putative active component 1-deoxynojirimycin (DNJ). Extracts of mulberry fruit and leaf (below) were used as sources of DNJ, supported by evidence of its inhibitory effect on alpha-glucosidase and impact of mulberry extracts on PPG in humans [21–23].
2. Mulberry leaf extract, 1.0 g (MLE; batch No. M0232060, Draco Natural Products Inc., San Jose CA, USA), containing 0.8% (w/w) DNJ.
3. Apple extract, 2.0 g (AE; Appl’in, batch No. 31083006, Diana Naturals, France) derived from apple press cakes, containing minimum 80% total polyphenols and 5% w/w/ of the putative key active component phloridzin (phlorizin). AE has previously been reported to lower PPG [24, 25] based on an established inhibitory effect of phloridzin on SGLT1 activity or possibly (also) GLUT2 inhibition derived from hydrolysis of phloridzin to its aglycone phloretin [26].
4. White Bean Extract, 3.0 g (WBE; extract of *Phaseolus vulgaris*, StarchLite, batch No. 9547/SO, Ingredia Nutritional, France). WBE has been reported efficacious for lowering PPG [27–31], an effect attributed to α-amylase inhibitory activity of one or more lectins or other (glyco) proteins [32].
5. Elderberry Extract 2.0 g (EE; batch No. L11IC03896, BerryPharma AG, Germany), containing 29.08% anthocyanins expressed as equivalents of cyanidin-3-glycoside (C3G). Flavonoid glycosides have been reported to act as SGLT1 inhibitors [8, 10, 33], but evidence for effects of EE or its components on glycemic control is largely derived from animal studies [34, 35], rather than any direct evidence of efficacy of EE or C3G for PPG reduction in humans.
6. Turmeric 0.18 g (TE; Curcumin C3 Complex, Sabinsa Europe GmbH, Germany, batch No. C110439), containing > 95% total curcuminoids & > 70% of the putative primary active component curcumin. Curcuminoids have been shown to reduce glucose absorption in rats, an effect presumed due to GLUT2 inhibitor activity [36, 37]. High-dose curcuminoids (300 mg/d, about 6–8 g turmeric) has been reported to improve several markers of glucose metabolism in diabetic humans [38, 39], though not found efficacious for reducing PPG [40].
7. AE (2.0 g) combined with TE (0.18 g). The combinations of TE with AE and TE with EE were included based on observations that combinations of extracts primarily inhibiting the SGLT1 (AE and EE) together with GLUT2 (TE) might have added functionality for slowing rates of glucose uptake [7, 41, 42].
8. EE (2.0 g) combined with TE (0.18 g), as described above.

The control and product used as the vehicle for intervention was a 60 g serving of the rice component of a commercial instant rice porridge mix (Knorr ‘cup Jok’; Unilever Thai Holdings Limited, Thailand), containing ~ 50 g available carbohydrate, 5 g protein, 1 g fat and 1 g fibre. Testing by a modified Englyst procedure [43] indicated that the proportions of rapidly digestible, slowly digestible and resistant starch in the carbohydrate component were ~ 84, ~ 16, and  < 1%, respectively. Each serving of rice was prepared by adding 300 ml of boiling water, allowing to cool to approximately 60 °C, and then stirring in the allocated test extract. There was no attempt to match products for sensory attributes or blind subjects to differences in colour and flavor. Subjects’ awareness of these differences were deemed unlikely to impact upon the physiological outcome measures, although self-reported symptoms might be affected. Alternative approaches to the delivery of extracts such as pills or intubation were judged likely to introduce effects on release, dissolution, and mixing that could much more directly impact the physiological measures of interest and their interpretation.

Subjects were instructed to consume the prepared rice with 200 ml of water, within a 15 min period. If any subject was not able to finish consuming the rice porridge within 15 min, the 15th minute blood sample was taken and they finished consuming the test product immediately after blood sampling. Time of consumption of test product was recorded. If any subject was unable to consume the full quantity of the test product, they would be excluded from the study.

Subjects were not allowed to consume any food after test product administration, except the lunch that was provided after the last blood sample had been collected. Subjects were not allowed to drink water 1 h prior to product administration. They were then allowed a maximum additional 500 ml water for the rest of the time until blood sampling was completed.

### Data and sample collection

#### Testing for lactase deficiency

As noted above, at the second screening visit, a breath hydrogen test was carried out to confirm lactase deficiency using the method and criteria of Satta et al. [44]. Subjects were required to fast overnight for at least 12 h before the test. Two consecutive baseline breath hydrogen measurements were made, prior to subjects ingesting 25 g lactose dissolved in 250 ml water. Breath hydrogen was then recorded every 30 min for the next 4 h (30, 60, 90, 120, 150, 180, 210 and 240 min) after the lactose intake. An increase in the breath hydrogen concentration of at least 20 ppm at any time point, after lactose ingestion, as compared to baseline sample, was considered as a positive test for lactase deficiency.

#### Blood draws

On test days, an intravenous indwelling cannula was inserted in a forearm vein of the subjects and 0.5 ml of normal saline solution injected to maintain the cannula patent for blood collection. Blood samples were collected after discarding the first 0.5 mL of normal saline containing blood from the tubing. Alternatively, if the cannula was blocked or there was difficulty in withdrawing blood through the cannula, blood samples could be withdrawn by a fresh vein puncture using a disposable sterile syringe and a needle at each time of collection. Two consecutive baseline blood samples, with a gap of maximum 5 min, were collected within a period of 15 min before the test product ingestion. From commencement of eating, subsequent samples were collected 15, 30, 45, 60, 90, 120, 180 min. The actual time of collection of each blood sample was recorded immediately after blood collection, and variation of ±1 min was considered acceptable for each time point of blood sampling. Time points outside this allowed range as above were documented as protocol deviations. In all such instances appropriate time corrections for the actual time of sample collection were incorporated at the time of data analysis.

#### Breath hydrogen sampling

Subjects exhaled into the breath hydrogen monitor at − 20, + 65, + 125, + 185, + 245, + 305, + 365 and + 425 min relative to ingestion of the test product.

#### Gastrointestinal discomfort and defecation self-reports

A questionnaire for gastrointestinal discomfort was completed by subjects on paper at baseline (before the blood sampling), + 2 h 10 m, + 4 h 10 m, and + 6 h 10 m minutes after test product consumption and at the end of each test day, and at + 24 h by interview. Intensity of nausea, flatulence, bloating and pain were rated as ‘none’ (= 0), ‘mild’ (=1), ‘moderate‘ (=2) or ‘severe’ (=3). Subjects were interviewed regarding the number and consistency of stools produced in the 24 h before study product intake (baseline), at the end of the test day, and 24 h after study product intake. Stool consistency was evaluated by the semi-quantitative Bristol scale (score between 1 = constipation and 7 = watery diarrhea) [45]. Colour illustrations of the stool chart were used in interviews and provided to each subject at the end of the first treatment visit to facilitate responding to the questionnaire by telephone.

A variation of ±5 min for breath sample analysis and questionnaire administration was considered acceptable and no protocol deviations were recorded within this allowed range.

#### Urine collection

Subjects were asked to empty their bladder before study product intake and before leaving the site. All urine produced during the test day was collected.

### Analytical procedures

All analyses were carried out by LTRL at the clinic test facility.

Breath hydrogen was directly measured in breath exhaled into a Gastrolyzer2™ breath hydrogen monitor (Bedfont Scientific Ltd., Maidstone, UK).

For plasma glucose and serum insulin, venous blood (5 ml) was collected into sodium fluoride tubes. A 3 ml aliquot was transferred to a tube with a clotting activator for measurement of serum insulin, followed by 2.0 ml into sodium fluoride tubes for plasma glucose. The blood samples were kept in the wet ice box for a maximum time of 30 min, and then centrifuged at 2500–3000 rpm for 10 min at ambient temperature to separate plasma. Proper clot formation was ensured before centrifugation for serum separation. Duplicate aliquots of plasma and serum were prepared for each end point were transferred within 15 min of separation for analysis (plasma glucose) or storage (serum samples), using gel packs to cool samples during transfer. One aliquot of plasma was analysed immediately and the other was stored at − 20 °C, for re-analysis if required. Two aliquots of serum for insulin were stored at − 20 °C immediately after collection.

Glucose was measured in fluoride plasma based on glucose oxidase-peroxidase principle by reflectance photometry (Vitros chemistry platform, Ortho Clinical Diagnostics, Raritan NJ, USA). Insulin was measured using an electrochemiluminescence immunoassay (Roche e411 assay and Elecsys 1010/2010 immunoassay analyzer, Roche Diagnostics GmbH, Mannheim, Germany).

For urine glucose testing, all urine samples voided while on site were tested using a dipstick yielding a semi-quantitative positive result (+, ++ or +++) for glucose above the detection limit of 2.8 mmol/L.

### Statistical analyses

Statistical analyses were carried out according to a pre-specified plan. No interim analyses were planned or performed, and treatment assignments were revealed only after blind review of the data following the clinical data collection phase. The blind review was undertaken by the principle investigator, statistician, and principle sponsor contact, and the treatment code was broken only after a hard lock of the data was agreed following this blind review.

The primary outcome measure was positive incremental area under the venous plasma glucose response over 2 h following ingestion of a treatment (+iAUC_2hr_). A power calculation indicated 62 subjects were needed for the intended design, based on an average + iAUC_2hr_ of 105 mmol.min/L, an effect size of 25, 80% power, 8 comparisons and an adjusted overall Type 1 error rate of 0.20. To balance the treatment sequences and accommodate anticipated dropouts, 72 subjects were recruited.

All available results from subjects were included in all analyses if they completed the control visit and at least one of the treatments. For the pre-specified analyses of glucose and insulin, data at all time points from baseline through 120 min were used, and the values at 180 min analyzed separately as an exploratory endpoint. If the glucose measurement was early or delayed, the target intended time, not the actual time, was used for calculations. Baseline glucose values were taken as the mean of the concentrations at times − 15 and 0 min. The iAUC_2hr_ for glucose was calculated using the trapezium method to obtain the total AUC (tAUC), and area below baseline subtracted to give iAUC. If any section of the curve moved below and then crossed back above baseline, the times marking the start and end of that section were obtained by linear interpolation and the area below the baseline added back to the iAUC to give +iAUC. The tAUC_2hr_ for insulin was calculated as described above for glucose tAUC.

The baseline was included as a covariate without regard to its degree of significance in all models that had a baseline measurement available, if the analysis method allowed. Body weight was also included in the models for glucose and insulin as weight determines relative carbohydrate load (g/kg). Statistical comparisons were only made between the control and the other test meals (i.e. eight statistical comparisons in total), with Dunnett’s test used to control the overall type 1 error rate. The primary glucose +iAUC_2hr_ endpoint was tested using a linear mixed model of the form +iAUC = baseline + subject_baseline + weight + visit + treatment + error, where baseline was the mean baseline value for that visit for that subject and subject_baseline was the mean baseline score over all visits for the subject. This latter term was included to avoid possible bias in the estimates of the treatment effect due to the use of a mixed model and the inclusion of a different baseline value at each visit. Visit was a categorical variable for the number of the visit (i.e. 3 to 7). The error terms were assumed to be normally distributed. Insulin AUC and other exploratory glucose and insulin parameters of interest were analysed using similar mixed models. Data in tables are presented as mean and standard error of the mean (SEM) adjusted for baseline.

For breath hydrogen, a reading of 10 ppm or more above the baseline value during the 7-h test period was regarded as a “positive” (physiologically relevant) increase in breath hydrogen [46]. The frequency proportion of subjects exhibiting an increase of 10 ppm above basal level at any time during the 420 min of follow-up was compared between each treatment and control. For gastrointestinal discomfort, the frequency of scores (0, 1, 2 and 3) were obtained for each of the 4 symptoms (nausea, flatulence, bloating and pain) at the interview 7 h after consumption of treatment. Only subjects whose score for a symptom was ≤1 at baseline were considered for analysis. As the incidence of scores > 0 was very low, only frequencies and proportions of high scores are reported.

For stool consistency, a score of 6 or 7 on the Bristol scale or > 3 stools during the past 24 h assessed at the 24 h interview was scored as diarrhoea. No statistical analysis was planned for glucose in urine, and any positive indication of glucose in a urine sample would have been recorded as an adverse event.

All reported *p*-values are two-sided, with *p* < 0.05 used as the criterion for statistical significance.

## Results

### Analysis population

Supplementary Figure S1 describes the flow of subject numbers through the recruitment, screening and intervention phases, and Table S3 shows the number of subjects randomized to receive and number completing each treatment. Of the 72 subjects starting the study, seven were completely excluded from the analysis because they only attended the first test visit (five due to vomiting, one unable to eat the test meal, and one for personal reasons) and provided no usable efficacy data. Of the remaining 65 subjects, one subject missed two visits for personal reasons, one subject missed the final visit due to a positive pregnancy test and two others vomited at their last visit. The dropouts and missing data were similarly spread across the active treatments (Table S3). None of these missed visits occurred when the control product was served, and the analysis was therefore based on all data available from these 65 subjects including those who missed 1 or 2 test sessions. Subjects were mean (SD) 31.2 (5.5) yr old, 159.0 (9.2) cm, 56.1 (8.7) kg, and BMI 22.1 (2.0) kg/m^2^.

### Efficacy and tolerance measures

Only the addition of extracts derived from AE, MFE and MLE produced statistically significant effects on the primary outcome PPG + iAUC_2hr_ (*p* < 0.05) compared to the control, and all of those reduced PPG by more than 20% (Table 1). The other treatments produced reductions smaller than 10% (or even a mean increase) in PPG, which were not statistically significant.

**Table 1:** Plasma glucose results, as positive incremental area under the curve over 2 h (+iAUC_2hr_, min.mmol/L)

Intervention	N	Mean adjusted for baseline	SEM	Mean % difference from control product (lower, upper adjusted 95% CI)	Adjusted p-value vs control product
AE	33	124.0	14.3	−25.4 (− 48.3,-2.5)	0.021
EE	30	156.9	14.9	−5.6 (− 29.2, 18.1)	0.996
MFE	34	106.7	14.0	−35.8 (−58.2, −13.4)	< 0.001
MLE	33	126.1	14.4	−24.1 (−47.1, −1.2)	0.033
TE	33	166.4	14.3	0.2 (−22.7, 23.0)	1.00
AE + TE	30	132.9	15.1	−20.1 (−44.2, 4.1)	0.163
EE + TE	31	151.3	14.7	−8.9 (−32.4, 14.6)	0.921
WBE	31	186.1	14.7	12.0 (−11.5, 35.5)	0.719
Control product	65	166.2	11.2		

AE, EE, MFE, MLE, TE, WBE are extracts of apple, elderberry, mulberry fruit, mulberry leaf, turmeric, and white bean, respectively. N, number of subjects receiving each extract (all subjects received the control product). SEM, standard error of the mean

With the exception of TE (alone) and WBE, all extracts and combinations had statistically significant (*p* < 0.01) effects on tAUC_2hr_ insulin, all of these exceeding a 15% reduction in responses relative to the control (Table 2).

**Table 2:** Serum insulin results, as total area under the curve for 2 h (tAUC_2hr_, min.mIU/L)

Intervention	N	Mean adjusted for baseline	SEM	Mean % difference from control product (lower, upper adjusted 95% CI)	Adjusted p-value vs control product
AE	32	5678	382	−22.3 (−35.3, −9.3)	<.001
EE	30	6040	389	−17.3 (−30.6, −4.1)	0.003
MFE	34	5087	371	−30.4 (−43.0, −17.8)	<.001
MLE	32	5190	383	−29.0 (−42.1, −15.8)	<.001
TE	32	7527	383	3.0 (−10.1, 16.1)	0.996
AE + TE	30	5400	395	−26.1 (−39.6, −12.6)	<.001
EE + TE	31	5781	389	−20.9 (−34.2, −7.6)	0.001
WBE	31	7820	389	7.0 (−6.2, 20.3)	0.681
Control product	65	7307	306		

AE, EE, MFE, MLE, TE, WBE are extracts of apple, elderberry, mulberry fruit, mulberry leaf, turmeric, and white bean, respectively. N, number of subjects receiving each extract (all subjects received the control product). SEM, standard error of the mean

Because mean breath H_2_ excretion decreased after test product intake and only started to rise again after 5 h, the mean + iAUC_0-7h_ values were highly skewed and could not be used for the pre-planned statistical analyses. The analysis of breath hydrogen was therefore based on the frequency of increases > 10 ppm above initial baseline (Table 3). Such rises in breath H_2_ production were infrequent, but higher in both mulberry extracts, though statistically more frequent than control only for MLE (*p* < 0.02). Additional *post-hoc*, exploratory analyses were carried out to establish possible relationships between measures of PPG and breath hydrogen. In addition to assessing the correlation between these measures, results were further split into ‘responders’ and ‘non-responders’ based on an increase of at least 10 ppm in breath hydrogen either from baseline or the minimum values between 0 and 120 min for all interventions. None of these relationships were statistically significant (all *p* > 0.10; data not shown).

**Table 3:** Frequency of subjects having a maximum breath hydrogen > 10 part per million (ppm) above baseline per treatment

Intervention	N	% subjects with maximum breath hydrogen > 10 ppm	p-value vs control product
AE	33	0	1.00
EE	30	0	1.00
MFE	34	17.7	0.14
MLE	33	24.2	0.02
TE	33	0	1.00
AE + TE	30	6.7	0.99
EE + TE	31	6.5	0.70
WBE	31	0	1.00
Control product	65	3.1	

AE, EE, MFE, MLE, TE, WBE are extracts of apple, elderberry, mulberry fruit, mulberry leaf, turmeric, and white bean, respectively. N, number of subjects receiving each extract (all subjects received the control product)

Scores for gastrointestinal discomfort were extremely low for all treatments, and no treatment effects could be discerned. Mild bloating was reported in 33 (3.6%) out of 926 completed questionnaires on gastrointestinal discomfort. Bloating was reported at least once for most treatments and varied from 0% (MLE) to 5.6% (EE, TE + AE). There were two reports of mild nausea (AE, WBE) and one for pain in the bowel (MLE). No flatulence was reported. No gastrointestinal discomfort was reported in the 320 interviews taken at the end of each treatment day. No cases of constipation or diarrhea were reported before (17 h), during (7 h) or after (24 h) the treatments. There were no occurrences of glucose in urine.

## Discussion

These data support the efficacy of the AE, MFE and MLE used here for the reduction of PPG and PPI in healthy subjects consuming a realistic carbohydrate load. Addition of these plant extracts significantly and substantially reduced PPG compared to the reference. In addition, except for TE (alone) and WBE, all extracts and combinations significantly and substantially reduced PPI.

The results also suggest generally good tolerance to all of the extracts at the doses used, with low indications of ‘off-target’ effects. Rises in breath H_2_ production > 10 ppm over baseline (an indicator of carbohydrate malabsorption) were infrequent, and statistically more frequent than reference only for MLE. Scores for gastrointestinal discomfort were extremely low for all treatments, with no apparent differences among them. Although it is possible that post-absorptive mechanisms could have contributed to the observed reductions in PPG, there were no indications of glucose losses in urine.

This research adds to a variable quality and quantity of evidence for each of the extracts tested.

The AE tested here showed significant efficacy for PPG and PPI, a result which adds to a limited and inconsistent body of research using various AE preparations. The same commercially available AE used here was stated to reduce PPG in unpublished studies cited in a European Food Safety Authority health claims application [47]. More recently, Castro-Acosta et al. [48] reported that this AE (1.8 g total, 151 mg phloridzin), consumed prior to a meal containing 41 g starch and 22 g sugars, produced a statistically significant reduction in PPG and PPI AUC responses over 30 min but not 2 h. However, the magnitude of mean reductions in 2-h PPG and PPI AUC were similar to those observed here. Johnston et al. [24] tested a different AE at lower levels of phloridzin (approximately 12.5 and 28 mg vs ~ 100 mg here) and reported that the higher dosage reduced PPG AUC over 90 min, with evidence of a transient reduction in PPI. Schulze et al. [49] reported that an AE with a considerably higher (448 mg) phloridzin level significantly reduced PPG and PPI at initial time points but not the overall 2 (or 3) hr. AUCs, and also significantly increased urinary glucose. While the AE tested here was standardized for phloridzin, different AEs may also contain variable levels of other polyphenols with potential bioactivity for the same or different targets. Therefore, variation in research results using other AE could reflect differences in the total composition of the extracts. Individual differences in the rates of hydrolysis of phloridzin to its aglycone phloretin could also contribute to variation in results among studies, although data from Blum et al. [50] suggest this would not have much impact. Other studies used European (and more likely lactose tolerant) subjects, whereas the present study specifically screened for lactose intolerance, which may improve the likelihood of observing a beneficial effect. In contrast to AE alone, the combination of AE + TE produced a somewhat smaller and non-significant reduction in PPG, but the reliability and explanation for this difference in outcome is uncertain.

We observed clear efficacious effects of MFE and MLE containing ~ 7.5–8 mg DNJ, for both PPG and PPI. In contrast to AE, there are many human studies that have reported on the acute efficacy of mulberry extracts, usually MLE [21, 51–60] for reducing PPG and, in fewer studies, also PPI. While different dose levels of DNJ have been tested within and between the published studies, almost all of these have found statistically significant reductions in one or both of these outcomes at some dose level. However, consistently significant effects for PPG and PPI have only been reported for MLE at doses containing ≥9 mg DNJ (vs ~ 6 mg or less, which generally were not efficacious).

EE had negligible effects on PPG, but relatively large and statistically significant effects on PPI. Despite the beneficial effects hypothesized from in vitro and animal research [34, 35], we found no human clinical data directly testing the effects of EE or other extracts rich in C-3G on PPG or PPI. It is therefore possible that a dose larger than that used here (2.0 g containing ~ 600 mg C3G) might have been more efficacious. However, two studies reported no significant effects of sustained consumption of EE containing 42 mg/d [61] or 500 mg/d [62] C3G on fasting glucose, while another trial reported that 640 mg/d of an anthocyanin mix containing mostly C3G had no effect on fasting insulin but raised fasting glucose levels [63]. Two other studies using blackcurrent [48] or blueberry [64] extracts with total anthocyanin levels similar to the EE used here reported variable effects on PPG and PPI at individual timepoints but no significant effect on AUC values over 2 or 2.5 h.

It is unclear why the EE tested here reduced PPI with no effect on PPG; however, the result seems to be robust, as it was almost identically observed for EE alone and for EE combined with TE (which by itself had little apparent physiological effect). An analogous result has been reported for a flavonoid-rich black tea, where the authors hypothesize that changes in PPI but not PPG may have been secondary to observed increases in peripheral vascular perfusion [65]. There are however no data to judge whether this might apply to EE, which has a very different composition. Other alternative mechanisms could include an effect on muscle glucose uptake [66] or perhaps release of incretins, but there is no supporting evidence for such effects in vivo.

Although WBE has a long history of promotion as a dietary supplement, the specific WBE used here, at the 3 g dose recommended by the supplier, had no apparent physiological effects under these test conditions. This is in contrast to a number of previous trials that have tested the effects of different sources and the same or lower doses of WBE on PPG relative to a placebo control. Louisa et al. [31] reported that 1.5 g of a WBE supplement following a rice meal produced a statistically significantly mean reduction of 9.5% in PPG AUC over 4 h. Vinson et al. [29] reported that 1.5 WBE mixed into margarine and eaten with ~ 60 g carbohydrate as bread significantly reduced PPG AUC over 80 min, by 66%. Similarly, Udani et al. [30] found that a 3 g doses of WBE as a powder mixed in margarine, or taken as a tablet, significantly reduced the PPG AUC over 2 h following a 50 g carbohydrate load as white bread. Using only 100 mg of a different WBE, Spadafranca et al. [28] reported evidence of reduced PPG and PPI responses to high-carbohydrate meal, with no significant adverse gastrointestinal effects. A range of other clinical data on WBE are variable in outcomes and quality, and somewhat difficult to interpret, due to differences in material specifications and research designs [27, 67–69]. That literature also indicates substantial inconsistencies in the reported outcomes, ranging from no effects at all up to large amounts of weight loss with sustained use [27], which might imply substantial inhibition of carbohydrate digestion accompanied by malabsorption. The specific, commercially-available WBE used here showed only marginal inhibition of alpha-amylase activity in our assays (see supplemental material), and limited water solubility, which could explain the lack of apparent efficacy.

Like WBE, the TE used here showed no apparent efficacy for PPG or PPI when given alone, nor did it have any added effects when combined with AE or EE. The hypothesized benefits of TE, especially in combination with AE or EE, were largely derived from theoretical considerations and evidence from in vitro assays [7, 41]. Empirical evidence for any beneficial effects of TE on PPG or PPI, or the doses or components required for efficacy, is limited. Wickenberg et al. [40] reported that 6 g of an unspecified turmeric preparation had no effect on PPG but significantly increased PPI following 75 g glucose. In contrast, Zanzer et al. [70] reported that a TE standardized to a total polyphenol concentration of 185 mg gallic acid equivalents significantly reduced PPG up to 45 min but not the AUC over 2 or 3 h following a 50 g carbohydrate meal, and had no significant effects on PPI. It is possible that the weak in vivo efficacy of the specific TE used here or in other research reflects insufficient doses or bioaccessibility of one or more putative functional curcuminoids [71].

There was no evidence of adverse gastrointestinal symptoms for any extract, and a statistically significant increase in breath H_2_ only for MLE. Other studies using MLE have also reported significant increases in H_2_ [53–56, 72]. However, no significant effects of MLE on gastrointestinal symptoms were apparent here or in other studies where these were assessed [52, 59, 72]. The overall reporting of such symptoms was overall very low in this study, although similar questionnaires have been found sensitive to various diet and drug treatments, including clinical studies specifically in India [73, 74]. Interestingly, with the same primary active agent and dose, MFE had somewhat greater efficacy for PPG and yet smaller effects on breath H_2_. There are no other comparable published data on MFE, but this result is consistent with our *post-hoc* analyses which found no significant association of PPG responses with breath H_2_.

There was also no evidence that any treatment produced glucosuria. We are not aware of other studies using these extracts where this has been assessed. This suggests that, for example, the dose of phloridzin in AE that was efficacious for lowering PPG and PPI, was below the dose where renal glucose reabsorption via SGLT2 might have been affected.

While this study produced fairly clear results in terms of efficacy, there are a number of factors that may limit their wider generalization. Most importantly, the results can only be assumed to apply to extracts with similar doses and specifications of the putative active components. The results were also achieved with extracts stirred into a semi-solid extruded rice composition primarily composed of rapidly digestible starch, so outcomes may differ with other carbohydrate sources or methods of food preparation. Lastly, we used an Indian population and included only lactase-deficient subjects, in order to minimize potential conversion of phloridzin to phloretin, and allow for a clear interpretation of the AE data. It is unlikely but possible that this could have improved the apparent efficacy of AE [50] or other extracts.

## Conclusions

This trial has demonstrated that additions of AE, MFE and MLE, with the specifications and doses used here, produced robust reductions of PPG and PPI in healthy subjects consuming a realistic carbohydrate load. In contrast, the EE, TE and WBE used here had no significant effects on PPG, although EE reduced PPI. All extracts were well tolerated, with no indications of gastrointestinal discomfort or glucosuria. Taken together, further testing and assessment of feasibility for commercial food use could be of value for the extracts which were consistently efficacious here. This would require that these effects can be reliably replicated, and that any products using these extracts would meet applicable technical, safety and consumer acceptance criteria. In addition, further research should address dose-response, efficacy in other food formats and in other populations including individuals with (pre-)diabetes, and ultimately perhaps also the sustained effects or impact on markers of disease risk.

## Supplementary information

**Additional file 1:** Background, specification and in vitro bioactivity of extracts used for intervention. **Table S1.** Inclusion and exclusion criteria. **Table S2.** Time and event schedule for volunteers **Figure S1.** Overview of subjects screened, randomized and entering the study **Table S3.** Number of subjects randomized to and completing treatment visits for each extract and the control product.

## Data Availability

The datasets generated during the current study are not publicly available as the participants did not give express consent for this, but are available from the corresponding author on reasonable request.

## Abbreviations

AE: Apple extract
AUC: Area under the curve (iAUC = incremental AUC; tAUC = total AUC)
BMI: Body Mass Index
C3G: Cyanidin-3-glycoside
CI: Confidence Interval
DNJ: 1-deoxynojirimycin
EE: Elderberry extract
GLUT2: Glucose transporter 2
H_2_: Breath hydrogen
LPH: Lactase-phloridzin hydrolase
LTRL: Lambda Therapeutics Research Ltd.
MFE: Mulberry fruit extract
MLE: Mulberry leaf extract
PPG: Post-prandial glucose
PPI: Post-prandial insulin
SD: Standard Deviation
SEM: Standard Error of the Mean
SGLT1: Sodium-glucose linked co-transporter 1
TE: Turmeric extract
WBE: White bean extract
