# Supplementary material for: The effect of 8 plant extracts and combinations on post-prandial blood glucose and insulin responses in healthy adults: a randomized controlled trial
Source: Nutr Metab (Lond). 2020 Jul 6;17:51. doi: 10.1186/s12986-020-00471-x (PMC7336677; doi:10.1186/s12986-020-00471-x)
Supplement: Supplementary file 1 — Additional file 1: Background, specification and in vitro bioactivity of extracts used for intervention. Table S1. Inclusion and exclusion criteria. Table S2. Time and event schedule for volunteers Figure S1. Overview of subjects screened, randomized and entering the study Table S3. Number of subjects randomized to and completing treatment visits for each extract and the control product. [file 12986_2020_471_MOESM1_ESM.docx]

**Supplementary text, tables and figures**

**Background, specification and *in vitro* bioactivity of extracts used for intervention**

1. Mulberry fruit extract (MFE) and Mulberry leaf extract (MLE)

The nature and sources of mulberry extracts, and the supporting evidence for their (*in vitro* and clinical) bio-efficacy and putative active constituents and mechanisms, have been widely reported (1). MFE and MLE are primarily used and standardized as sources of 1-deoxynojirimycin (DNJ), a known α-glucosidase inhibitor, although they contain additional iminosugars and other constituents with potentially relevant biological activity (2, 3). Efficacy of DNJ and mulberry (especially leaf) extracts for reducing PPG and PPI is supported by a relatively large published body of *in vitro*, animal and human clinical studies (3-6).

The MFE and MLE (batch No. MF-DC-KQ and M0232060, respectively, Draco Natural Products Inc. USA) used in clinical testing contained 0.5% (w/w) and 0.8% (w/w) DNJ, respectively, as determined by high performance liquid chromatography with an evaporative light scattering detector. The *in vitro* efficacy of both extracts was assessed in an alpha-glucosidase assay by using p-nitrophenyl-alpha- glucopyranoside as the substrate. Acarbose was tested as positive control with an IC_50_ of 11.8 μg/mL. Both MFE and MLE showed potent inhibitory activities against alpha-glucosidase, with IC_50_ values of 2.6 and 2.9 μg/ml, respectively.

The MFE was also tested in a Caco-2 GLUT2 glucose transporter assay using radiolabelled 2-deoxyglucose as substrate. MFE inhibited GLUT2-mediated glucose uptake with an IC_50_ of 1.5 mg/ml (assuming the extract did not contain any D-glucose). Pure DNJ did not inhibit GLUT2 glucose transport at concentrations ranging from 0.5-5 µM. Phloridzin (IC_50_ for SGLT1 ~0.3 µM = 0.13 mg/l), phloretin (IC_50_ for GLUT2 ~80 µM = 22 mg/l) and dapagliflozin (IC_50_ for SGLT1 ~0.5 µM = 0.2 mg/l) were used as positive controls in all assays

1. Apple extract (AE)

The AE (Appl’in, Batch No. 31083006, Diana Naturals, France) used in clinical testing was derived from apple press cakes (7, 8) and contained >80% total polyphenols (catechin equivalents) and >5% Phloridzin (phlorizin). Phloridzin is known to inhibit SGLT1 and has shown clinical efficacy for PPG lowering (9). In addition, AE may also exhibit putative GLUT transporter inhibition, including possible GLUT2 inhibition derived from other components as well as *in vivo* hydrolysis of phloridzin (via lactase phloridzin hydrolase) to its aglycone phloretin (9, 10). There are limited human clinical data supporting the potential PPG-lowering efficacy of the AE. This includes published and unpublished evidence on the AE used here (7, 11) and mixed data for effects on PPG and PPI from studies using AEs with a different composition (12, 13).

The *in vitro* efficacy of AE was assayed in a polarised Caco-2 glucose transport assay at both low (5mM, 15 min) and high (25mM, 30 min) glucose concentrations. Phloridzin and phloretin were tested as positive controls, for low glucose (predominantly SGLT1-driven transport) and high glucose (predominantly GLUT2-driven transport) respectively. Evidence of SGLT1 inhibition was defined as >20% inhibition at 5mM glucose and ≤20% inhibition at 25mM glucose, and GLUT2 inhibition as >20% inhibition at both 5mM & 25mM glucose. See Mohammad et al. (14) for additional details.

Phloridzin (300uM), Phloretin (150uM) & AE (400 ug/ml) demonstrated 52%, 84% & 60% inhibition of glucose transport respectively under low glucose conditions and 8%, 76% & 63% inhibition of glucose transport respectively under high glucose conditions.

AE was furthermore tested *in vitro* for SGLT1 and GLUT2 inhibitory properties using a Caco-2 cell intestinal glucose uptake model and radiolabelled glucose analogues as substrates. AE at 0.2 mg/ml inhibited 90% of SGLT1 activity with an IC_50_ of 0.45 mg/ml for GLUT2 inhibition (assuming the AE did not contain D-glucose). Phloridzin (IC_50_ SGLT1 ~0.3 µM = 0.13 mg/l), phloretin (IC_50_ GLUT2 ~80 µM = 22 mg/l) and dapagliflozin (IC_50_ SGLT1 ~0.5 µM = 0.2 mg/l) were used as positive controls in all assays. The AE results are indicative of the presence of both SGLT1 and GLUT2 inhibitors within the extract.

1. White Bean Extract (WBE)

Extracts of white bean (*Phaseolus vulgaris*) are widely sold commercially as food supplements and ingredients with claims of efficacy as an inhibitor of starch digestion. The α-amylase inhibitory activity is attributed in many literature sources to ‘phaseolamin’, a term variously applied to one or more lectins or other (glyco)proteins (15-17). However, in other literature a clear distinction is made between phaseolamin (suggested to be a storage protein) and the lectin-like α-amylase inhibitors (18, 19). While WBE has well-known *in vitro* α-amylase inhibitor activity, the lack of clarity on the nature and terminology of the functional agent in WBE means different commercial materials are standardized for α-amylase inhibitor activity (15) rather than a specific chemical profile. Several studies have reported efficacy of different WBE sources and doses for PPG-lowering, although the wider body of data on WBE conveys inconsistent physiological effects (19-26). This may reflect the different compositions and study designs, small study populations, but also uncontrolled trials or trials using WBE within a combination of treatments.

The *in vitro* activity of WBE (StarchLite, Ingredia International, France, batch No. 9547/SO) was evaluated on both porcine and human pancreatic alpha-amylase assays by using alpha-(2-Chloro-4- nitrophenyl)-beta-1,4-galactopyranosylmaltoside as a substrate. Acarbose was tested as the positive control with IC_50_ values of 1.1 μg/ml and 1.4 μg/ml against porcine and human alpha-amylase, respectively. WBE showed limited water solubility (<1 mg/ml) and relatively low inhibition of alpha-amylase from both human and porcine pancreas (less than 10% inhibition at 200 μg/ml).

1. Elderberry Extract (EE)

EE was selected as a concentrated food-grade source of cyanidin-3-glycoside (C3G). Flavonoid glycosides in general and CSG specifically have been reported to be (possibly selective) SGLT1 inhibitors with mixed evidence for efficacy in reducing PPG (27-29). An anthocyanin-rich extract of elderberry has also been reported to have some α-glucosidase inhibitory activity (30). To our knowledge, EE or its putative active C3G have never been directly tested for PPG-lowering efficacy. However, in a study of the effects of acute and repeated elderberry juice consumption on blood lipids, Murkovic et al. (31) incidentally reported no changes in blood glucose levels.

EE Powder (Batch No. L11IC03896, BerryPharma AG, Germany) used in clinical testing contained 29.08% Anthocyanins (expressed as C3G equivalents). EE was tested in the polarised Caco-2 glucose transport assay as described above for AE, and with the same controls. EE (200ug/ml) demonstrated 22% and 17% inhibition of glucose transport respectively under low and high glucose conditions. The EE results are indicative of SGLT1 inhibition.

1. Turmeric (TE)

TE was selected as a source of curcuminoids, which have been shown to reduce glucose absorption in rats, an effect presumed due to GLUT2 inhibitor activity (32). Lin and Chen (33) reported that curcumin blocked hepatic GLUT2 expression and membrane translocation. There are few clinical data on the PPG effects of turmeric, TE or curcuminoids. Despite promising results in animals, Wickenberg et al. (34) reported that 6 g turmeric (approximately 200-300 mg curcuminoids) had no effect on PPG following a 75 g glucose load, while significantly increasing insulin responses. Zanzer et al (35) reported significant effects of a TE on early PPG timepoints, but not on the AUC over 2 or 3 hours. An insulinogenic effect has been suggested by *in vitro* work with pancreatic beta cells (36). In addition to positive evidence from animal research, sustained use of high-dose curcuminoids (300 mg/d, about 6-8 g turmeric) has also been reported to improve several markers of glucose metabolism in diabetic humans (37, 38). However, those authors generally suggest post-absorptive targets rather than a gut-mediated PPG-lowering effect as the underlying mechanism.

The TE (Curcumin C3 Complex, batch No. C110439, Sabinsa Europe GmbH, Germany) used in clinical testing contained >95% total curcuminoids & >70% curcumin. The *in vitro* efficacy of curcumin extract was assayed in a polarised Caco-2 glucose transport assay as described above for AE, and with the same controls. TE (300uM) demonstrated 68% and 70% inhibition of glucose transport respectively under low and high glucose conditions. The TE results are indicative of GLUT2 inhibition.

1. Combinations of AE+TE and of EE+TE

These combinations were included based on observations that combinations of extracts primarily inhibiting the SGLT1 (AE, EE) and GLUT2 (TE) transporters together may have significant added functionality for slowing rates of glucose uptake (39-41).

References

1. Andrade JF, Silva V, Melnik T. Mulberry therapy for type 2 diabetes mellitus. Cochrane Database Syst Rev. 2015(5):DOI: 10.1002/14651858.CD011697.

2. Oku T, Yamada M, Nakamura M, Sadamori N, Nakamura S. Inhibitory effects of extractives from leaves of *Morus alba* on human and rat small intestinal disaccharidase activity. Br J Nutr. 2006;95(05):933-8.

3. Kimura T. Development of mulberry leaf extract for suppressing postprandial blood glucose elevation. In: Rigobelo E, editor. Hypoglycemia - Causes and Occurrences. Rijeka, Croatia: INTECH Open Access Publisher; 2011. p. 25-36.

4. Phimarn W, Wichaiyo K, Silpsavikul K, Sungthong B, Saramunee K. A meta-analysis of efficacy of Morus alba Linn. to improve blood glucose and lipid profile. Eur J Nutr. 2017;56(4):1509-21.

5. Shin S-O, Seo H-J, Park H, Song HJ. Effects of mulberry leaf extract on blood glucose and serum lipid profiles in patients with type 2 diabetes mellitus: A systematic review. Eur J Integrative Med. 2016;8(5):602-8.

6. Tian S, Tang M, Zhao B. Current anti-diabetes mechanisms and clinical trials using *Morus alba* L. J Trad Chin Med Sci. 2016;3(1):3-8.

7. EFSA. Scientific opinion on the substantiation of a health claim related to “Appl'In® polyphenolic apple extract powder (*Malus domestica*)” and reduction of post-prandial glycaemic responses pursuant to Article 13(5) of Regulation (EC) No 1924/2006. EFSA J. 2011;9(10).

8. Personal communication with supplier.

9. Ehrenkranz JRL, Lewis NG, Ronald Kahn C, Roth J. Phlorizin: a review. Diabetes Metab Res Rev. 2005;21(1):31-8.

10. Besnard M, Megard D, Rousseau I, Zaragoza MC, Martinez N, Mit Javila MT, et al. Polyphenolic apple extract: Characterisation, safety and potential effect on human glucose metabolism. Agro Food Ind Hi Tech. 2008;19(4):16-9.

11. Castro-Acosta ML, Stone SG, Mok JE, Mhajan RK, Fu C-I, Lenihan-Geels GN, et al. Apple and blackcurrant polyphenol-rich drinks decrease postprandial glucose, insulin and incretin response to a high-carbohydrate meal in healthy men and women. J Nutr Biochem. 2017;49:53-62.

12. Johnston KL, Clifford MN, Morgan LM. Possible role for apple juice phenolic compounds in the acute modification of glucose tolerance and gastrointestinal hormone secretion in humans. J Sci Food Agric. 2002;82(15):1800-5.

13. Schulze C, Bangert A, Schwanck B, Vollert H, Blaschek W, Daniel H. Extracts and flavonoids from onion inhibit the intestinal sodium-coupled glucose transporter 1 (SGLT1) in vitro but show no anti-hyperglycaemic effects in vivo in normoglycaemic mice and human volunteers. J Funct Foods. 2015;18:117-28.

14. Mohammad RH, Nur-e-Alam M, Lahmann M, Parveen I, Tizzard GJ, Coles SJ, et al. Isolation and characterisation of 13 pterosins and pterosides from bracken (Pteridium aquilinum (L.) Kuhn) rhizome. Phytochemistry. 2016;128:82-94.

15. Boniglia C, Carratù B, Di Stefano S, Giammarioli S, Mosca M, Sanzini E. Lectins, trypsin and α-amylase inhibitors in dietary supplements containing *Phaseolus vulgaris*. Eur Food Res Technol. 2007;227(3):689-93.

16. Mosca M, Boniglia C, Carratù B, Giammarioli S, Nera V, Sanzini E. Determination of α-amylase inhibitor activity of phaseolamin from kidney bean (*Phaseolus vulgaris*) in dietary supplements by HPAEC-PAD. Anal Chim Acta. 2008;617(1-2):192-5.

17. Savelkoul FHMG, Van Der Poel AFB, Tamminga S. The presence and inactivation of trypsin inhibitors, tannins, lectins and amylase inhibitors in legume seeds during germination. A review. Plant Foods Hum Nutr. 1992;42(1):71-85.

18. Moreno J, Altabella T, Chrispeels MJ. Characterization of α-amylase-inhibitor, a lectin-like protein in the seeds of *Phaseolus vulgaris*. Plant Physiol. 1990;92(3):703-9.

19. Obiro WC, Zhang T, Jiang B. The nutraceutical role of the *Phaseolus vulgaris* α-amylase inhibitor. Br J Nutr. 2008;100(01):1-12.

20. Barrett ML, Udani JK. A proprietary alpha-amylase inhibitor from white bean (*Phaseolus vulgaris*): A review of clinical studies on weight loss and glycemic control. Nutr J. 2011;10(1):DOI: 10.1186/475-2891-10-24.

21. Onakpoya I, Aldaas S, Terry R, Ernst E. The efficacy of *Phaseolus vulgaris* as a weight-loss supplement: a systematic review and meta-analysis of randomised clinical trials. Br J Nutr. 2011;106(02):196-202.

22. Ulbricht C, Bryan JK, Conquer J, Costa D, Stock T, Tanguay-Colucci S, et al. An evidence-based systematic review of amylase inhibitors by the natural standard research collaboration. J Diet Suppl. 2010;7(1):78-95.

23. Louisa M, Arozal W, Widjaja I, Setiawaty A, Soetikno V. Phaseolus vulgaris Extract Reduced Glucose Absorption After Rice Intake: A Study in Indonesian Healthy Volunteers. Advanced Science Letters. 2017;23(7):6889-92.

24. Spadafranca A, Rinelli S, Riva A, Morazzoni P, Magni P, Bertoli S, et al. *Phaseolus vulgaris* extract affects glycometabolic and appetite control in healthy human subjects. Br J Nutr. 2013;109(10):1789-95.

25. Udani JK, Singh BB, Barrett ML, Preuss HG. Lowering the glycemic index of white bread using a white bean extract. Nutr J. 2009;8(1):52.

26. Vinson JA, Kharrat HA, Shuta D. Investigation of an amylase inhibitor on human glucose absorption after starch consumption. Open Nutraceuticals Journal. 2009;2(1).

27. Johnston K, Sharp P, Clifford M, Morgan L. Dietary polyphenols decrease glucose uptake by human intestinal Caco-2 cells. FEBS Lett. 2005;579(7):1653-7.

28. Nicolle E, Souard F, Faure P, Boumendjel A. Flavonoids as promising lead compounds in type 2 diabetes mellitus: Molecules of interest and structure-activity relationship. Curr Med Chem. 2011;18(17):2661-72.

29. Castro-Acosta ML, Lenihan-Geels GN, Corpe CP, Hall WL. Berries and anthocyanins: promising functional food ingredients with postprandial glycaemia-lowering effects. Proc Nutr Soc. 2016;75(03):342-55.

30. Matsui T, Ueda T, Oki T, Sugita K, Terahara N, Matsumoto K. α-Glucosidase inhibitory action of natural acylated anthocyanins. 1. Survey of natural pigments with potent inhibitory activity. J Agric Food Chem. 2001;49(4):1948-51.

31. Murkovic M, Abuja PM, Bergmann AR, Zirngast A, Adam U, Winklhofer-Roob BM, et al. Effects of elderberry juice on fasting and postprandial serum lipids and low-density lipoprotein oxidation in healthy volunteers: a randomized, double-blind, placebo-controlled study. Eur J Clin Nutr. 2004;58(2):244-9.

32. Sivabalan S, Anuradha CV. A comparative study on the antioxidant and glucose-lowering effects of curcumin and bisdemethoxycurcumin analog through *in vitro* assays. Int J Pharmacol. 2010;6(5):664-9.

33. Lin J, Chen A. Curcumin diminishes the impacts of hyperglycemia on the activation of hepatic stellate cells by suppressing membrane translocation and gene expression of glucose transporter-2. Mol Cell Endocrinol. 2011;333(2):160-71.

34. Wickenberg J, Ingemansson SL, Hlebowicz J. Effects of *Curcuma longa* (turmeric) on postprandial plasma glucose and insulin in healthy subjects. Nutr J. 2010;9(1).

35. Zanzer YC, Plaza M, Dougkas A, Turner C, Björck I, Östman E. Polyphenol-rich spice-based beverages modulated postprandial early glycaemia, appetite and pyy after breakfast challenge in healthy subjects: A randomized, single blind, crossover study. J Funct Foods. 2017;35:574-83.

36. Best L, Elliott AC, Brown PD. Curcumin induces electrical activity in rat pancreatic β-cells by activating the volume-regulated anion channel. Biochem Pharmacol. 2007;73(11):1768-75.

37. Na L-X, Li Y, Pan H-Z, Zhou X-L, Sun D-J, Meng M, et al. Curcuminoids exert glucose-lowering effect in type 2 diabetes by decreasing serum free fatty acids: a double-blind, placebo-controlled trial. Mol Nutr Food Res. 2012;57(9):1569-77.

38. Gupta SC, Patchva S, Aggarwal BB. Therapeutic roles of curcumin: Lessons learned from clinical trials. AAPS J. 2013;15(1):195-218.

39. Scow JS, Iqbal CW, Jones TW, Qandeel HG, Zheng Y, Duenes JA, et al. Absence of evidence of translocation of GLUT2 to the apical membrane of enterocytes in everted intestinal sleeves. J Surg Res. 2011;167(1):56-61.

40. Berry MJ, Fowler MI, Heath AD. Edible composition. WO 2012168108 A1; 2012.

41. Farrell TL, Ellam SL, Forrelli T, Williamson G. Attenuation of glucose transport across Caco‐2 cell monolayers by a polyphenol‐rich herbal extract: Interactions with SGLT1 and GLUT2 transporters. Biofactors. 2013;39(4):448-56.

**Table S1. Inclusion and exclusion criteria.**

Subjects who met the following criteria could be included in the study:

1. Willing and able to give consent to participate in the study in writing
2. Age >20 and <50 yr
3. Body Mass Index (BMI) >18 and <25 kg/m^2^
4. Lactase deficient as indicated by screening test (see methods)
5. Volunteer apparently healthy [No medical conditions which might affect study measurement, as judged by study physician or measured by questionnaire, and/or assessed by haematology, blood chemistry and urinalysis]
6. Willing to comply to study protocol
7. Agree to be informed by study physician about medically-relevant personal test results
8. Willing to refrain from drinking alcohol the day of and one day before blood withdrawals
9. Fasting blood glucose value >3.4 and <6.1 mmol/ litre (62-110 mg/dl)
10. Haemoglobin level within normal reference range as judged by the research physician
11. Literate

Subjects who met any of the following criteria were excluded from the study:

1. Employee of Unilever, Hindustan Lever, or Lambda Therapeutics Research
2. Participated in any other biomedical study within 3 months before screening visit day for this study and/or participating in any other biomedical study during the study period
3. Alcohol intake > 120 ml/week
4. On a medically prescribed/slimming diet
5. Work in night shifts (between 23.00 and 6.00 hrs) in the week preceding or during the study
6. Use of any medication including traditional medicines, vitamins or tonics which might interfere with study measurements, as judged by the PI and/or study physician?
7. Engaging in intense exercise >10h/week? (defined as exercise which induces sweating and causes sufficient breathlessness to limit conversation)
8. Reported weight loss/gain >10% of body weight in the 6 months preceding screening
9. Blood donation within 2 months prior to screening visit
10. Evidence of drug abuse based on urine analysis
11. Allergy to any food or cosmetics
12. Smoking or consumption of tobacco in any form, and/or was smoking or consuming tobacco in any form within 6 months preceding the study and/or smoking or consuming tobacco in any form, during the study
13. Pregnant or planning pregnancy during the study period
14. Lactating or has been lactating within 6 weeks before pre-study investigation and/or during the study period

**Table S2. Time and event schedule for volunteers**

Screening Visit 1

Age, identity and literacy proof

1^st^ informed consent form (for screening)

Weight, height, physical examination and medical history

Alcohol breath and drug abuse test

Urine pregnancy test

Blood sampling (8 ml for fasting blood glucose and other screening tests)

Lunch

Screening Visit 2

Verification of identify

Instructions for lactose intolerance test

Lactose Intolerance test:

Baseline breath hydrogen (2 consecutive readings)

Lactose intake (25 gm in 250 ml water)

Breath hydrogen measurement (30, 60, 90, 120 min after lactose)

2^nd^ informed consent form (ICF-2, for study participation of lactose intolerant subjects)

Instructions for subsequent steps

Intervention Visits (Visits 3 to 7; each visit 2 days plus day 3 telephone follow-up)

*Day 1 (Check-in day)*

Arrive on site ~17:00

Verification of identity

Health and compliance check

Alcohol breath test

Pregnancy test

Allocation to wards

Dinner with standardized meals* (20:00-21.00 hr)

*Day 2 (Treatment day)*

Baseline urine and dipstick for glucose (within -60 minutes)

Questionnaire on 24 hr history stool consistency & frequency (-45 Minutes)

Baseline gastrointestinal symptoms questionnaire (-35 Minutes)

Baseline breath hrdrogen test (-20 minutes)

Fasting blood samples (2 samples at -15 to 0 min)

Test product intake (0 to +15 min)

Blood sampling (+15, 30, 45, 60, 90, 120, and 180 min)

Breath hydrogen test (+65, 125, 185, 245, 305, 365, and 425 min)

GI symptom questionnaire (+130, 250, 370 Minutes)

Collect and test for glucosuria on all spontaneously voided urine samples

Instruction to subjects for wash out period and for next visit

Questionnaire on stool consistency & frequency during test day (+440 min)

Collect mandatory urine sample for glucosuria test

Provide illustrated stool chart to refer to in Day 3 telephone call

Check-out from the facility

*Day 3 (Telephone call only)*

Questionnaire on stool consistency & quantity

*Subjects were provided with a standardized dinner to consume between 20.00 and 21.00 on the evening preceding all five treatment days. The actual quantity of food consumed by each subject prior to their initial treatment visit was recorded, and on subsequent visits, the same quantity of this identical meal was served to the subject. Subjects were not permitted to take any other food or beverage, except water, after dinner, until the test meal was provided after baseline samples were taken in the morning of each treatment day.

**Figure S1:** Overview of subjects screened, randomized and entering the study

Screened

N=253

Excluded based on other inclusion and exclusion criteria N=71

Invited for lactose intolerance test

N=182

Excluded on negative lactose intolerance test

N=46

Successfully screened N=136

Excluded by lot

N=63

Randomly invited for participation N=73

Dropped out before start of study N=1

Randomized to study N=72

**Table S3**: Number of subjects randomized to and completing treatment visits for each extract and the control product. All subjects were randomized to a sequence consisting of the control product and 4 of the 8 extracts. AE, EE, MFE, MFE, MLE, TE, WBE are extracts of apple, elderberry, mulberry fruit, mulberry leaf, turmeric, and white bean, respectively.

| Treatment | | | Number of subjects randomized to treatment | | | Number of subjects completing treatment | | |  |  |
| --- | --- | --- | --- | --- | --- | --- | --- | --- | --- | --- |
| AE | | | 37 | | | 33 | | |  |  |
| EE | | | 35 | | | 30 | | |  |  |
| MFE | | | 37 | | | 34 | | |  |  |
| MLE | | | 37 | | | 33 | | |  |  |
| TE | | | 37 | | | 33 | | |  |  |
| AE + TE | | | 35 | | | 30 | | |  |  |
| EE + TE | | | 35 | | | 31 | | |  |  |
| WBE | | | 35 | | | 31 | | |  |  |
| Control product | | | 72 | | | 65 | | |  |  |
|  | |  |  | |  |  | |  |  | |
|  | |  |  | |  |  | |  |  | |
|  | |  |  | |  |  | |  |  | |
|  | |  |  | |  |  | |  |  | |
|  | |  |  | |  |  | |  |  | |
|  | |  |  | |  |  | |  |  | |
|  | |  |  | |  |  | |  |  | |
|  | |  |  | |  |  | |  |  | |
|  | |  |  | |  |  | |  |  | |
|  | |  |  | |  |  | |  |  | |
